# SARS-CoV-2 Spike protein S2 subunit modulates γ-secretase and enhances amyloid-β production in COVID-19 neuropathy

**DOI:** 10.1038/s41421-022-00458-3

**Published:** 2022-09-30

**Authors:** Guanqin Ma, Deng-Feng Zhang, Qing-Cui Zou, Xiaochun Xie, Ling Xu, Xiao-Li Feng, Xiaohong Li, Jian-Bao Han, Dandan Yu, Zhong-Hua Deng, Wang Qu, Junyi Long, Ming-Hua Li, Yong-Gang Yao, Jianxiong Zeng

**Affiliations:** 1 Key Laboratory of Animal Models and Human Disease Mechanisms of the Chinese Academy of Sciences, and KIZ-CUHK Joint Laboratory of Bioresources and Molecular Research in Common Diseases, Kunming Institute of Zoology, Chinese Academy of Sciences, Kunming, Yunnan China; 2 Kunming National High-level Biosafety Research Center for Non-Human Primates, Center for Biosafety Mega-Science, Kunming Institute of Zoology, Chinese Academy of Sciences, Kunming, Yunnan China; 3 Kunming College of Life Science, University of Chinese Academy of Sciences, Kunming, Yunnan China; 4 CAS Center for Excellence in Brain Science and Intelligence Technology, Chinese Academy of Sciences, Shanghai, China; 5 Yunnan Key Laboratory of Biodiversity Information, Kunming Institute of Zoology, Chinese Academy of Sciences, Kunming, Yunnan China

**Keywords:** Mechanisms of disease, Transcriptomics

Dear Editor,

SARS-CoV-2-induced multi-lineage neural cell dysregulation has been documented^1^. SARS-CoV-2 infection elevates neuroinflammation^2^, alters brain structure^3^ leads to abnormal accumulation of neurodegenerative amyloid-β (Aβ) and phosphorylated tau^4,5^, and increases the risk of cognitive impairment^6^ in COVID-19 patients. However, the mechanism underlying neurological dysfunctions following SARS-CoV-2 infection remains largely unknown.

To evaluate long-term impact of SARS-CoV-2 infection to the brain, the hACE2 transgenic mice as described previously^7^ were intranasally infected with a single low dose (1 × 10^2^ TCID_50_) of prototyped SARS-CoV-2 and maintained for up to 30 days post infection (dpi) (Fig. 1a). Presence of SARS-CoV-2 was found in cortex at 7 dpi but not at 30 dpi by the viral Spike protein immunostaining (Supplementary Fig. S1a). We found a remarkable activation of Iba1^+^ microglia and GFAP^+^ astrocytes in the hippocampus and cortex of infected mice at 30 dpi (Supplementary Fig. S1b–e), suggesting a persistent neuroinflammation. We looked for further brain changes by analyzing transcriptomics of the hippocampal tissues at 30 dpi (Supplementary Fig. S2a). A series of dysregulated genes or pathways were identified in response to SARS-CoV-2 infection (Supplementary Table S1). Gene ontology analysis revealed that the upregulated genes were mainly enriched in pathways related to antiviral immune response and aging, while the downregulated genes were enriched in neuronal function-related pathways such as synaptic vesicle clustering (Fig. 1b). Specifically, the neuroinflammatory genes *Trem2*, *Ifitm3* and *Gfap* were significantly upregulated, whereas the neuronal genes *Map2* and *Synapsin II* (*Syn2)* were downregulated. Unexpectedly, mRNA levels of amyloid precursor protein (APP) processing-related genes such as *Bace1*, *Aph1*, *Presenilin 1* (*Psen1*), *Nicastrin* (*Ncstn*), and *Psenen* were unchanged (Fig. 1c). The upregulation of *Trem2* and *Gfap*, the downregulation of *Map2* and *Syn2*, and the un-alteration of *Bace1* and *Psen1* were validated by quantitative real-time PCR (Supplementary Fig. S2b). Such expression patterns were also observed in the brain transcriptomic dataset obtained from COVID-19 patients by single-nucleus RNA sequencing^2^ (Supplementary Fig. S3a–c). These results suggest that the presence of the neurodegenerative hallmarks in COVID-19 brain might not be regulated at the transcriptional level but through an unknown regulatory mechanism.

**Fig. 1:**
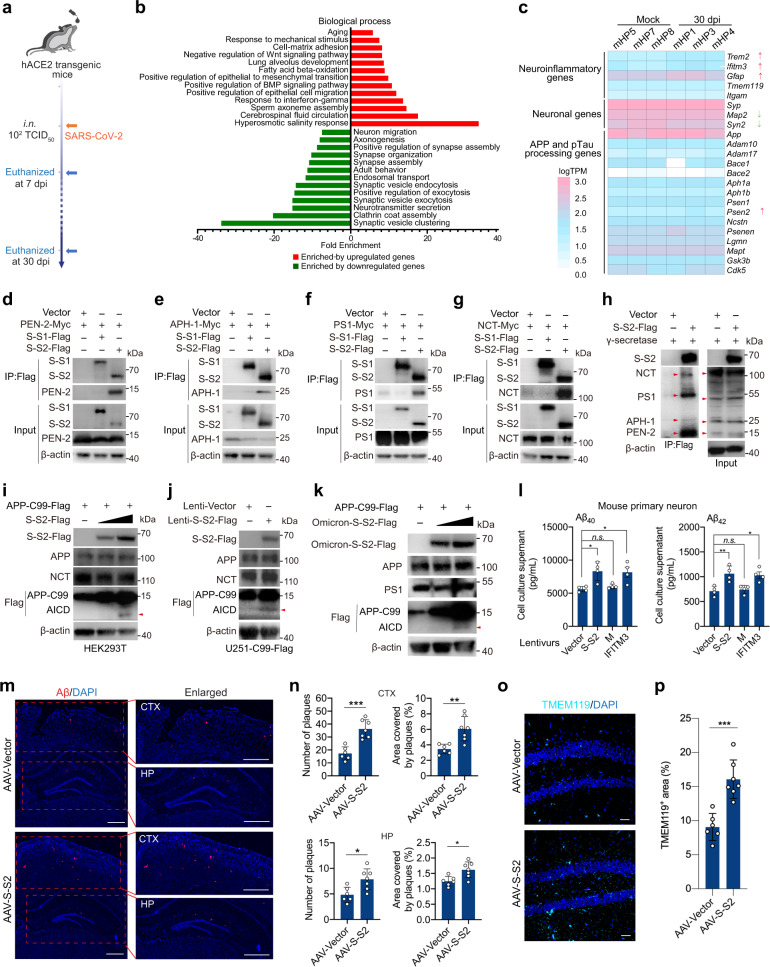
SARS-CoV-2 Spike protein S2 subunit binds to and modulates γ-secretase to enhance Aβ production. **a** hACE2 transgenic mice were intranasally (i.n.*)* infected by prototyped SARS-CoV-2. Brain cortical or hippocampal tissues were collected for immunofluorescence (7 or 30 dpi) and RNA-seq analysis (30 dpi). **b** Enrichment analysis of representative biological processes in the hippocampal RNA-seq data at 30 dpi in **a**. **c** Expression pattern of representative genes within the categorized gene ontology as indicated. **d**–**g** co-IP assays of anti-flag monoclonal antibody in HEK293T cells transfected with vector, S-S1-flag or S-S2-flag, together with myc-tagged PEN-2 (**d**), APH-1 (**e**), PS1 (**f**), and NCT (**g**). **h** co-IP assays of anti-flag monoclonal antibody in HEK293T cells co-transfected with myc-tagged PEN-2, APH-1, PS1 and NCT, together with S-S2-flag. **i** HEK293T cells were transfected with expression vector of APP-C99 with C-terminal flag tag (0.5 μg) and increasing amount of prototyped S-S2-Flag (0, 0.25, and 0.5 μg) in 12-well plates for 36 h. **j** U251-C99 cells were transduced with lentivirus carrying prototyped S-S2-Flag in 12-well plates for 36 h. The production of AICD (red arrows) in **i** and **j** was examined by immunoblot analysis. **k** HEK293T cells were co-transfected with expression vector of APP-C99 with C-terminal flag tag (APP-C99-Flag, 0.5 μg) and increasing amount (0, 0.25 or 0.5 μg) of Omicron S-S2-Flag in 12-well plates for 36 h. The production of AICD (red arrow) from APP-C99 was detected by immunoblot analysis. **l** Mouse primary neurons were isolated from embryonic (E18.5) brains and cultured in 24-well plates. Neurons were transduced with lentivirus carrying empty vector (vector), prototyped S-S2, M, or IFITM3 for 36 h. The Aβ40 (left) and Aβ42 (right) levels in the supernatants were quantified by ELISA. Means ± SD; *n* = 4; n.s., not significant; *P < 0.05; **P < 0.01, one-way ANOVA with Bonferroni’s post hoc test. **m** Representative anti-Aβ antibody staining of cortical (CTX) and hippocampal (HP) sections in APP/PS1ΔE9 mice with AAV delivery of prototyped S-S2 (AAV-S-S2) and AAV control (AAV-Vector). Scale bar, 500 μm. **n** Quantitative analysis of the number of Aβ plaques and the percentage of area covered by Aβ plaques in cortical (upper) and hippocampal (bottom) tissues in **m**. Each slide was counted for Aβ plaque number and Aβ plaque area via ImageJ software, and the percentage of the plaque area was calculated. **o** Representative immunofluorescence of microglial marker TMEM119 protein in hippocampal sections of AAV-S-S2 or AAV-Vector. Scale bar, 30 μm. **p** Quantification of percentage of TMEM119^+^ area in **o**. Statistical analyses for **n** and **p**, Means ± SD; *n* = 6 (AAV-Vector group) or *n* = 7 (AAV-S-S2 group); *P < 0.05; **P < 0.01*;* ***P < 0.001, Student’s *t*-test.

To explore potential mechanisms underlying COVID-19-related neuropathology, we tested whether SARS-CoV-2 membrane protein plays a role in this process. The γ-secretase complex, comprising PEN-2, APH-1, PS1 and NCT, is a critical membrane complex contributing to Aβ production in Alzheimer’s disease (AD) pathogenesis^8^. Initially, we conducted co-immunoprecipitation (co-IP) in HEK293T cells and found that SARS-CoV-2 Spike S2 subunit (S-S2), but not S-S1 protein, interacted individually with PEN-2 (Fig. 1d), APH-1 (Fig. 1e), PS1 (Fig. 1f) and NCT (Fig. 1g), and even bound to all these four components (Fig. 1h). The inverse co-IP could validate the interactions between S-S2 and PS1 or NCT (Supplementary Fig. S4a, b). To determine whether C-terminal transmembrane™ domain in S-S2 constitutes the structural basis for its interaction with γ-secretase, we examined membrane (M) protein of SARS-CoV-2 but found no interaction with PEN-2 and PS1 (Supplementary Fig. S4c, d), suggesting a specific interaction between S-S2 and γ-secretase. We next performed glutathione s-transferase (GST) pull-down and found that S-S2 can directly bind to PS1 and NCT (Supplementary Fig. S4e, f). Immunocytochemistry assay showed the co-colocalization of S-S2 with γ-secretase components individually in Hela cells (Supplementary Fig. S4g–j) and in the brain sections of infected mice at 7 dpi (Supplementary Fig. S4k).

SARS-CoV-2 Omicron variant (BA.1) Spike S2 subunit possesses six mutations (N764K, D796Y, N856K, Q954H, N969K, and L981F) compared to the prototype^9^. To see whether these mutations would interfere with its interaction with γ-secretase, co-IP assay in HEK293T cells showed that Omicron S-S2 not only interacted efficiently with PS1 and NCT (Supplementary Fig. S5a, b), but also had a comparable binding capacity to PS1 and NCT as prototyped S-S2 (Supplementary Fig. S5c, d), suggesting Omicron BA.1 S-S2 is capable of binding to γ-secretase.

An enzymatic cleavage of the APP by both β-secretase and γ-secretase, acting together, produces Aβ, which can cause widespread neuropathy within brain and is a pathological hallmark of AD^10^. The cleavage site of γ-secretase is located on C-terminal APP, namely APP C-terminal 99 fragment (APP-C99) only contains the cleavage site of γ-secretase. As a result, APP intracellular domain (AICD) at C-terminal C99 domain is produced by γ-secretase cleavage^11^. To examine whether the interaction between S-S2 and γ-secretase modulates the cleavage activity, we initially detected the production of AICD. Immunoblot showed that prototyped S-S2 promoted the production of flag-tagged AICD, whereas the expression of APP and NCT was largely unchanged (Fig. 1i). This was validated by the observation of the increased production of flag-tagged AICD in U251-C99 cells while the expression of APP and NCT was largely unaltered (Fig. 1j). Similarly, Omicron S-S2 also increased the production of flag-tagged AICD, while the expression of APP and PS1 was unchanged (Fig. 1k). These results demonstrate that the increased production of AICD from the APP cleavage was caused by S-S2 modulation of γ-secretase.

HEK-APP695^12^ cells transfected with prototyped S-S2, but not the M, produced higher level of Aβ40 than non-transfected cells via enzyme-linked immunosorbent assay (ELISA), while a similar increase of Aβ40 was also observed upon the transfection of IFITM3 as a positive control^13^ (Supplementary Fig. S6a). To further evaluate this effect, we used neuronal cells including U251 and mouse primary neurons, both endogenously expressing APP protein. Lentiviral transduction of prototyped S-S2 or IFITM3 invariably caused the increase of Aβ40 or Aβ42 production as compared to empty-vector lentivirus transduction in U251 cells (Supplementary Fig. S6b) and mouse primary neurons (Fig. 1l), whereas lentiviral transduction of the M did not have such an effect. As expected, mouse primary neurons transduced with lentiviral Omicron-S-S2 produced higher Aβ40 and Aβ42 levels (Supplementary Fig. S6c). These results demonstrate that SARS-CoV-2 Spike S2 subunit can modulate γ-secretase to increase Aβ production.

To investigate whether S-S2 modulates γ-secretase in vivo, we examined hippocampal and cortical tissues of APPswe/PSEN1dE9 (hereafter referred to as APP/PS1ΔE9) mice, which have mutated human APP (Swedish mutations K595N/M596L) and the human PSEN1/PS1 lacking exon 9^14^, 2 months after AAV delivery of S-S2. Immunohistochemistry showed a widespread overexpression of S-S2 in hippocampal tissues (Supplementary Fig. S7a). Measurement of soluble and insoluble Aβ levels using ELISA showed that soluble Aβ42 species, but not insoluble Aβ40 and Aβ42 and soluble Aβ40, were markedly increased in cortical tissues of APP/PS1ΔE9 mice with S-S2 overexpression relative to empty vector group (Supplementary Fig. S7b–e). Similarly, immunostaining showed a significant increase of Aβ burden in cortical and hippocampal tissues of APP/PS1ΔE9 mice after S-S2 delivery (Fig. 1m). The delivery of S-S2 increased the Aβ plaque-deposited area in cortical and hippocampal tissues of APP/PS1ΔE9 mice (Fig. 1n). Overall, overexpression of SARS-CoV-2 S-S2 in hippocampus exacerbated Aβ burden in APP/PS1ΔE9 mice.

Neuroinflammation, an important factor in AD pathogenesis, promotes Aβ pathology^15^. A significant increase of Iba1^+^ microglia and GFAP^+^ astrocytes (Supplementary Fig. S8a–c) was observed in hippocampal tissues of APP/PS1ΔE9 mice after delivery of S-S2. Staining of microglial marker TMEM119 validated the elevated neuroinflammation following S-S2 delivery (Fig. 1o, p). These results demonstrated that S-S2 overexpression increased Aβ deposit and caused neuroinflammation in Aβ pathology of APP/PS1ΔE9 mice. Both the area covered by NeuN-labeled neuronal cells and the thickness of NeuN-labeled CA1 subfield (Supplementary Fig. S8d–f) were not significantly altered in hippocampal tissues following S-S2 delivery, suggesting that S-S2 overexpression might not cause neuronal loss after AAV delivery for 2 months.

In summary, we have identified S-S2 subunit as a γ-secretase modulatory protein and revealed a previously unknown mechanistic insight into COVID-19-related neuropathological sequelae (Supplementary Fig. S9). A systematical examination of multiple Omicron sub-variants (Supplementary Fig. S10) on potential brain dysfunction would be inspired in future studies. The Spike protein could function as an immune switch to increase γ-secretase activity and Aβ production and contribute to neurological changes in COVID-19 patients.

## Supplementary information

Supplementary Information

Supplementary Table 1

Supplementary Table 2

Supplementary Table 3

## Data Availability

The hippocampal RNA-seq data were deposited in the NCBI GEO database under the accession number GSE199545.
