# Supplementary material for: SARS-CoV-2 Spike protein S2 subunit modulates γ-secretase and enhances amyloid-β production in COVID-19 neuropathy
Source: Cell Discov. 2022 Sep 30;8:99. doi: 10.1038/s41421-022-00458-3 (PMC9524330; doi:10.1038/s41421-022-00458-3)
Supplement: Supplementary file 1 — Supplementary Information [file 41421_2022_458_MOESM1_ESM.docx]

**Supplementary Information**

**SARS-CoV-2 Spike protein S2 subunit modulates γ-secretase and enhances amyloid-β production in COVID-19 neuropathy**

Guanqin Ma^1,6^, Deng-Feng Zhang^1,2,3,6^, Qing-Cui Zou^2,6^, Xiaochun Xie^1,6^, Ling Xu^1,2,3^, Xiao-Li Feng^2^, Xiaohong Li^1^, Jian-Bao Han^2^, Dandan Yu^1,2,3^, Zhong-Hua Deng^2^, Wang Qu^2^, Junyi Long^2^, Ming-Hua Li^2,*^, Yong-Gang Yao^1,2,3,4,*^, Jianxiong Zeng^1,2,3,5,*^

^1^Key Laboratory of Animal Models and Human Disease Mechanisms of the Chinese Academy of Sciences, and KIZ-CUHK Joint Laboratory of Bioresources and Molecular Research in Common Diseases, Kunming Institute of Zoology, Chinese Academy of Sciences, Kunming, Yunnan 650201, China

^2^Kunming National High-level Biosafety Research Center for Non-Human Primates, Center for Biosafety Mega-Science, Kunming Institute of Zoology, Chinese Academy of Sciences, Kunming, Yunnan 650107, China

^3^Kunming College of Life Science, University of Chinese Academy of Sciences, Kunming, Yunnan 650204, China

^4^CAS Center for Excellence in Brain Science and Intelligence Technology, Chinese Academy of Sciences, Shanghai 200031, China

^5^Yunnan Key Laboratory of Biodiversity Information, Kunming Institute of Zoology, Chinese Academy of Sciences, Yunnan, 650201, China

^6^These authors contributed equally

*Corresponding authors:

Ming-Hua Li (limh@mail.kiz.ac.cn)

Yong-Gang Yao (yaoyg@mail.kiz.ac.cn)

Jianxiong Zeng (zengjianxiong@mail.kiz.ac.cn)

**Materials and Methods**

**Cells**

HEK293T, Vero E6, Hela, and U251 cells were obtained from the Kunming Cell Bank, Kunming Institute of Zoology (KIZ), and were grown in Dulbecco's Modified Eagle medium (DMEM) high glucose supplemented with 10% FBS, 1% penicillin/streptomycin at 37°C in humidified 5% CO_2_ and 95% air. Mouse primary neurons were isolated and cultured as described previously^1^. Briefly, cerebral cortices from E18.5 mouse embryos were dissected, carefully stripped of their meninges, digested with TrypLE Express Enzyme (Catalog # 12604013; ThermoFisher Scientific) with RQ1 RNase-free DNase (Catalog # M6101; Promega) for 20 min at 37°C, and dispersed to single-cell level by passing through a 70 µm cell strainer. The cell suspension was then cultured with Neurobasal medium supplemented with B27 Supplement (Catalog # 17504044; ThermoFisher Scientific) at 37°C in humidified 5% CO_2_, 95% air on poly-D-Lysine (Catalog # P0899; Millipore) pre-coated coverslips in 12-well culture plates. Half the medium was replaced on alternate days.

**Virus**

The SARS-CoV-2 strain was kindly provided by Guangdong Provincial Center for Disease Control and Prevention, Guangdong Province of China and was described in our previous studies^2,3^. The virus was propagated and titrated in Vero E6 cells, which were cultured in DMEM supplemented with 2% FBS. The viral sequence is accessible in the China National Microbiology Data Center (Accession No. NMDCN0000HUI).

**Plasmids**

Human presenilin-1 (PS1), nicastrin (NCT), anterior pharynx-defective 1 (APH-1), or presenilin enhancer 2 (PEN-2) were cloned into pCAGGS vector with a C-terminal myc tag. Prototyped SARS-CoV-2 full-length spike, Spike S1 subunit (S-S1), Spike S2 subunit (S-S2), membrane (M), Omicron BA.1 lineage Spike S2 subunit (Omicron S-S2) and human IFITM3 were cloned into pCAGGS vector with a C-terminal flag tag. Human APP695, APP-C99, prototyped SARS-CoV-2 Spike S1 subunit, Spike S2 subunit, and Omicron Spike S2 subunit were cloned lentiviral pCDH-CMV-MCS-EF1-Puro vector with a C-terminal flag tag. Prototyped SARS-CoV-2 Spike S2 subunit was also cloned into bacterial pGEX-6p-1 vector with a N-terminal GST tag.

**Glutathione-S-transferase (GST) pull-down**

For expression and purification of recombinant GST-fusion proteins, GST-S-S2 and the negative control GST protein were expressed in *E. coli* strain BL21 (DE3) at 25°C in the presence of 0.5 mM Isopropyl β-D-1-thiogalactopyranoside (IPTG). Cell pellets were resuspended in GST lysis buffer (150 mM NaCl, 1mM EDTA [pH 8.0], 20 mM Tris-Cl [pH 8.0] supplemented with 1% Triton X-100 and protease inhibitor cocktail (Catalog # K1007; Apexbio), followed by sonication, and the cell debris was removed by centrifugation (13,500 rcf at 4°C) for 30 min. The proteins in the supernatant were purified by glutathione Sepharose (Catalog # HY-K0234; MedChemExpress) accordingly to the manufacturer’s protocol and the purified proteins were quantified by a UV-visible spectrophotometer and the BCA protein assay kit (Catalog # P0010S; Beyotime). Purified GST-fusion proteins with the same amount were incubated with HEK293T cell lysates overnight and were then washed with the GST lysis buffer five times followed by Western immunoblotting analysis.

**Co-immunoprecipitation**

Cultured cells were lysed with RIPA lysis buffer (Catalog # P0013C; Beyotime) supplemented with 1% CHAPSO (Catalog # C3649; Millipore) and protease inhibitor cocktail (Catalog # K1007; Apexbio). After clarification and preclearing, protein amounts were quantified by the BCA protein assay kit (Catalog # P0010S; Beyotime). Around 1/10 volume of cell lysates was heated as whole cell lysates in Western blot analysis and the remaining 9/10 volume of the lysates was incubated with anti-flag magnetic beads (Catalog # HY-K0207; MedChemExperss) or anti-myc magnetic beads (Catalog # HY-K0206; MedChemExperss) for 16 h at 4°C. Immune complexes were washed with the lysis buffer and subjected to Western blot analysis.

**γ-secretase activity measurement**

HEK293T-APP695, U251 cells, or mouse primary neurons were co-transfected with expression vector for prototyped S-S2, M, Omicron S-S2, or IFITM3, or transduced with lentiviral prototyped S-S2, M, or Omicron S-S2. It should be noted that U251^4^ and primary neurons^5^ endogenously express APP. The cell culture supernatants were collected for enzyme-linked immunosorbent assay (ELISA) at 36 h post transfection or transduction. HEK293T-C99 were transfected with increased amount expression vector of prototyped S-S2 or Omicron S-S2, and U251-C99 cells were transduced with lentiviral prototyped S-S2. The resulting cell lysates were collected for Western blot at 36 h post transfection or transduction.

The resulting supernatants from cell cultures were collected and the concentrations of Aβ40/Aβ1-40 and Aβ42/Aβ1-42 were determined by using human Aβ40 kit (Catalog # E-EL-H0542c; Elabscience), human Aβ42 kit (Catalog # E-EL-H0543c; Elabscience), mouse Aβ40 kit (Catalog # E-EL-M3009; Elabscience), and mouse Aβ42 kit (Catalog # E-EL-M3010; Elabscience), respectively, following the manufacturer’s protocols.

**RNA-sequencing of hippocampal tissues from mouse model of SARS-CoV-2 infection**

We performed RNA-sequencing (RNA-seq) of hippocampal tissues from mice with and without SARS-CoV-2 infection. Total RNA (1.5 μg) per sample was used for the library preparation. Sequencing libraries were constructed using the NEBNext Ultra^TM^ RNA Library Prep kit for Illumina (NEB, USA) following the manufacturer’s instructions. Libraries were sequenced on an Illumina Nova seq platform and 150 bp paired-ends reads were generated. Sequencing reads were processed and differential gene expression analysis was performed according to standard protocols as described in our previous studies^6,7^. In brief, the raw reads were trimmed to remove sequencing adapters and low-quality reads with Trimmomatic^8^. The clean reads were aligned to the standard mouse reference genome (GRCm38) using STAR^9^. RSEM^10^ was then used to count aligned reads that mapped to the annotated mouse genes. Gene-level differential expression analyses were performed using R package DESeq2^11^. Gene Ontology biological processes enrichment analysis for differentially expressed genes was performed using clusterProfiler^12^.

**Cell type specific transcriptomic analysis of** **Aβ processing genes using single-nucleus RNA-seq data from cortex tissues of COVID-19 patients**

To investigate whether the Aβ processing genes were altered in cortical tissues of patients with COVID-19, and whether the differential expression was cell-type specific, we retrieved one reported single-cell RNA-seq dataset. The dataset contains 38,217 single-nucleus gene expression profiles from the medial frontal cortex of 8 controls and 8 COVID-19 patients^13^, with normalized counts data; and was downloaded from https://twc-stanford.shinyapps.io/scRNA_Brain_COVID19. The data was processed using Seurat^14^ and a violin plot was generated for each gene of interest grouped by cell-types and infection status.

**Animal models**

8-12 weeks old male hACE2 transgenic mice were purchased from the Shanghai Model Organisms as described previously^15^. After being anesthetized with isoflurane (RWD Life Science), the mice were intranasally infected with a total of 20 μL containing 1 × 10^2^ median tissue culture infectious Dose (TCID_50_) of live SARS-CoV-2 in the animal biosafety level 3 laboratory (ABSL-3) of the KIZ. At the indicated time points post infection, tissue samples were collected from the animals after euthanasia and stored in -80°C freezer (for RNA extraction) or in 4% paraformaldehyde (PFA; for immunofluorescence).

Animals were maintained on a 12-h light/dark cycle, with free access to food and water. All these experiments with live SARS-CoV-2 were performed in the ABLS3 laboratory of the KIZ. The Institutional Animal Care and Use Committee of KIZ approved all experimental procedures and protocols used in this study (Approval No. IACUC-RE-2021-05-009).

**Stable cell lines**

HEK293T or U251 cells stably overexpressing APP695 or APP-C99 were generated by lentiviral transduction. Briefly, the lentivirus was made by co-transfection of lentiviral transfer vector carrying APP695 (GenBank: A33292.1) or APP-C99 coding sequence (pCDH-CMV-MCS-EF1-Puro) and packaging plasmids pMD2G (Catalog # 12259; Addgene) and psPAX2 (Catalog # 12260; Addgene) into HEK293T cells using Lipofectamine 3000 (Catalog # L3000015; ThermoFisher Scientific). The lentivirus-containing supernatants were collected and pooled at 72 h post-transfection. HEK293T or U251 cells were transduced by the lentivirus at 1:1 dilution of culture medium and lentivirus-containing supernatant in the presence of 8 μg/mL polybrene (Millipore). The stable cells overexpressing APP695 or APP-C99 were selected and maintained in growth medium with 1 μg/mL puromycin (InvivoGen).

**Quantitative real-time PCR (qRT-PCR)**

Total RNA was extracted from homogenized brain tissues using a TRIzol reagent (ThermoFisher Scientific). Target transcripts were determined by qRT-PCR using qScriptTM One-Step qRT-PCR kit (Catalog # 95057-050; Quanta Biosciences) on CFX96 real-time PCR system (Bio-Rad). The primer sequences are listed in Supplementary Table 2.

**Immunofluorescence**

Immunofluorescence was performed as described in our previous studies^15,16^. Briefly, brain slides between bregma -1.8 mm and -2.2 mm were prepared via frozen sections for each animal. For antigen retrieval, slides were immersed in a quick antigen retrieval solution (P0090, Beyotime). Then slides were washed with 1 × phosphate-buffered saline (PBS; pH 7.4), and blocked with 5% bovine serum albumin (BSA) in 1 × PBST (0.3% Triton-X 100 in PBS) at 37°C for 60 min. The primary antibodies used are listed in Supplementary Table 3. The primary antibodies above were diluted in 3% donkey serum) in 1 × PBST (0.2% Triton-X 100) and incubated overnight at 4°C. Slides were then washed, and immunoreactivity was detected using Donkey anti-Rabbit IgG Highly Cross-Adsorbed Secondary Antibody, Alexa Fluor Plus 488, Donkey anti-Mouse IgG Highly Cross-Adsorbed Secondary Antibody, Alexa Fluor Plus 555, Donkey anti-Rat IgG Highly Cross-Adsorbed Secondary Antibody, Alexa Fluor Plus 647 (1:500; ThermoFisher Scientific) for 1 h at room temperature. Slides were counterstained with 5 μg/mL 4’,6-diamidino-2-phenylindole (DAPI; ThermoFisher Scientific) for 10 min at room temperature and washed with 1 × PBST (0.2% Triton-X 100) three times. Slides were visualized using ZEISS LSM 880 confocal microscope. The digital images were imported into ImageJ software in which the area of targeted signals was automatically analyzed, and the percentage of target signal was calculated.

**Immunoblotting**

Immunoblotting was performed as described previously^15,17^. In brief, protein samples were separated by SDS-PAGE and transferred to PVDF membrane by semi-dry transfer at 25 V for 30 min. The membrane was blocked in 5% skim milk in 1 × PBST for 1 h and incubated overnight with commercial primary antibody in 5% bovine serum albumin (BSA) at 4°C. The membrane was incubated with anti-mouse or anti-rabbit HRP-conjugated secondary antibodies in 5% milk and bands were developed with Chemi-Doc XRS imaging (Bio-Rad). The primary antibodies used are listed in Supplementary Table 3.

**Adenovirus associated virus (AAV) mediated gene delivery and tissue analyses**

5-month-old APP/PS1ΔE9 mice^18^ were used for AAV-mediated gene delivery as described previously^19^. Briefly, the recombinant AAV php.eb vectors, with GFP expression carrying empty vector (AAV-Vector) or SARS-CoV-2 Spike S2 subunit (AAV-S-S2) were developed. The purified viruses were stored at -80°C and diluted with saline to 1 × 10^13^ vector genomes (vg)/mL for injection. The mice were anesthetized by intraperitoneal injection of Zoletil-50 (80 mg/kg body weight) with xylazine (20 mg/kg body weight) and positioned on a stereotactic frame (Panlab, Harvard, MA, USA), then each animal was bilaterally injected with 1 μL viral solution (equivalent to 1 × 10^10^ vg) into the hippocampus (stereotaxic coordinates: anteroposterior, -2 mm; mediolateral, ±2.1 mm; dorsoventral, -1.9 mm) with a syringe pump (Panlab, Harvard, MA, USA) at a speed of 200 nL/min. The needle was left in place for an additional 5 min before being slowly removed. The mice were maintained at the experimental animal core facility of KIZ on a 12-h light/dark cycle, with free access to food and water. The effects of AAV-Vector and AAV-S-S2 on neurological outcomes in the mice were assessed at 2 months post injection. The animals were euthanized prior to the collection of brain tissues. Briefly, the brain was carefully removed and rinsed in cold PBS, followed by immediate dissection into two halves. A part of cortex was stored at -80°C for the biochemical assays, whereas the rest of the tissues were fixed in 4% paraformaldehyde in PBS at 4°C for the immunofluorescence assays.

We isolated plaque-related insoluble and soluble Aβ using the protocols described in our previous studies^19,20^. In brief, each sample of cortical tissue was weighed and then homogenized with 100 μL of RIPA lysis buffer (Catalog # P0013; Beyotime) containing protease inhibitor (Catalog # K1007; Apexbio) and phosphatase inhibitors (Catalog # HY-K0023; MedChemExpress) on ice, followed by centrifugation at 13,000 g for 15 min at 4°C, before collecting the supernatants and pellets. The protein concentrations of the supernatants were measured by using the BCA protein assay kit (Catalog # P0010S; Beyotime) and were adjusted to the same concentration for quantifying the soluble Aβ concentration by ELISA. The pellets were resuspended in a volume of 100 μL of RIPA lysis buffer, followed by centrifugation at 13,000 g for 15 min at 4°C to remove potential soluble Aβ. This washing step was repeated 3 times to remove all soluble Aβ. Then, the pellets containing insoluble Aβ were solubilized in sodium dodecyl sulfate (SDS) buffer (2% SDS, 25 mM Tris-HCl, pH 7.4). After pulsed sonication for 15 sec, the SDS fractions were quantified for protein concentrations and processed for measuring insoluble Aβ concentration, using the same procedure for soluble Aβ. The levels of Aβ40 and Aβ42 in mouse cortex tissues were determined by using the Aβ40 kit (Catalog # E-EL-H0542c; Elabscience) and Aβ42 kit (Catalog # E-EL-H0543c; Elabscience), respectively, following the manufacturer’s protocols. Although a recent event challenged the association between Aβ and Alzheimer’s disease^21^, the involvement of Aβ species Aβ40 and Aβ42 as indicators as neural cell dysregulation has not been questioned^22-25^. Therefore, we used the levels of Aβ40 and Aβ42 as a valid indicator for potential degeneration of brain during the development of Alzheimer’s disease.

**Statistical analysis**

All appropriate data were analyzed using GraphPad Prism 8 (GraphPad Software Inc.). All hypothesis tests were performed as two-tailed tests. Specific statistical analysis methods were described in the related figure legends where results are presented. Values were considered statistically significant for *p* values < 0.05.

**Supplementary Figures**

**
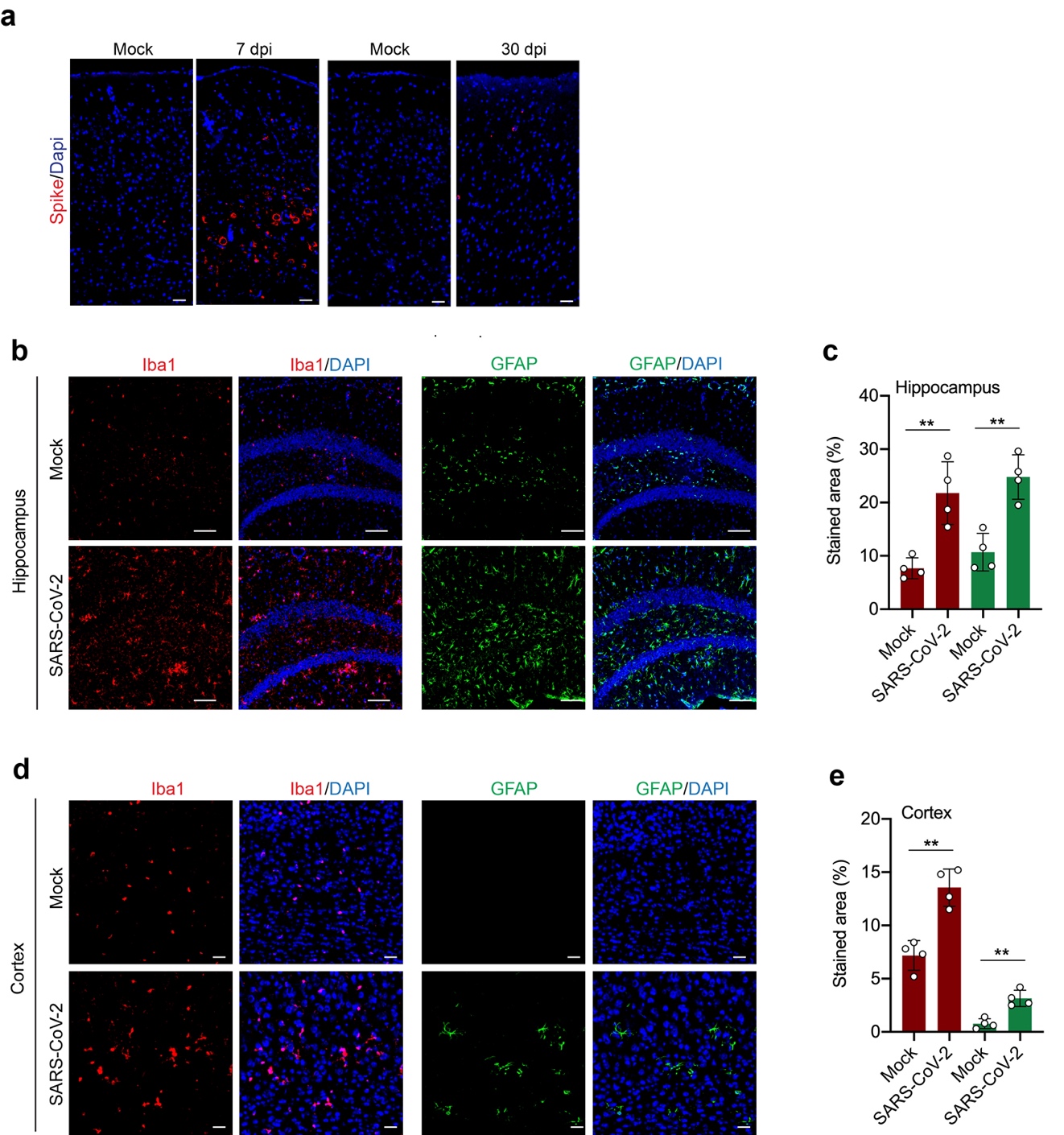
**

**Fig. S1 Activation of glial cells in brain sections of SARS-CoV-2-infected hACE2 transgenic mice**

**a** Representative immunofluorescence of SARS-CoV-2 Spike protein in cortical sections from infected mice at 7 dpi and 30 dpi and uninfected (mock) mice at the corresponding time points. Scale bar, 30 μm. **b** Representative immunofluorescence of microglial Iba1 (*left*) and astrocytic GFAP (*right*) of hippocampal area in mouse brain sections at 30 dpi. Scale bar, 30 μm. **c** Quantification of percentage of Iba1^+^ (red) and GFAP^+^ (green) area in **b**. Each slide was used and counted for stained area via ImageJ software, and the percentage of average of stained area each section was calculated. **d** Representative immunofluorescence of microglial Iba1 (*left*) and astrocytic GFAP (*right*) proteins of cortical area in mouse brain sections at 30 dpi. Scale bar, 30 μm. **e,** Quantification of percentage of Iba1^+^ (red) and GFAP^+^ (green) area in **d**. Statistical analysis in **c** and **e**, Mean ± SD; *n* = 4; **, *p* < 0.01, Student’s *t*-test.


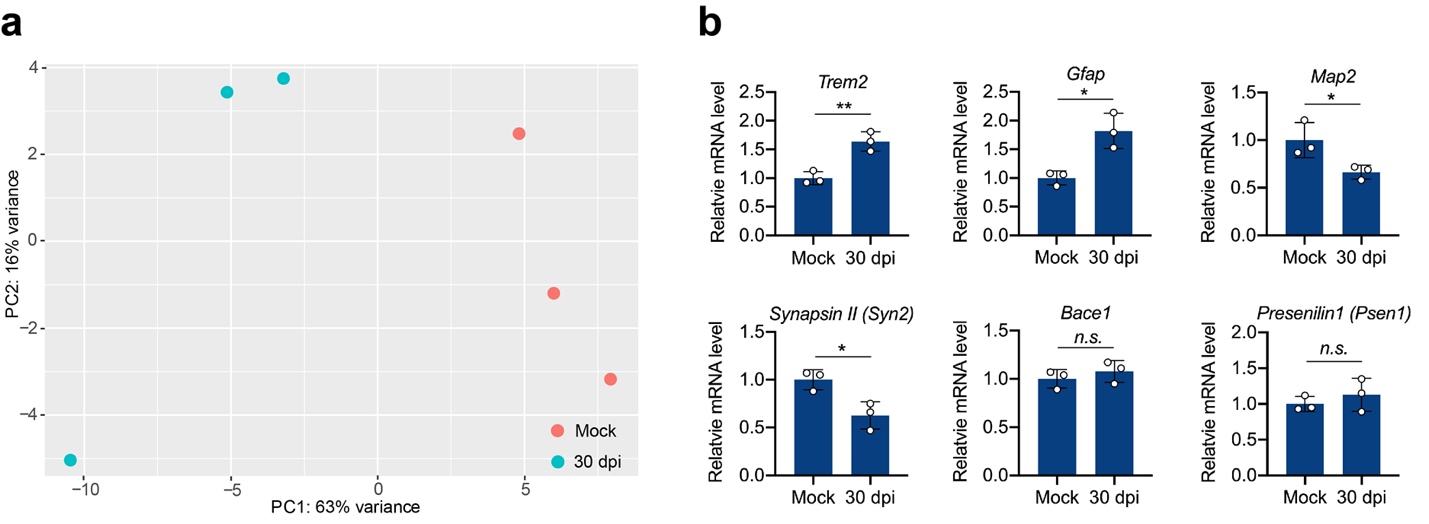


**Fig. S2 Quantification of the mRNA expression in SARS-CoV-2 infected brain hippocampal tissues**

**a** Principal component analysis of hippocampal samples from SARS-CoV-2 infected mice at 30 dpi (blue dot, *n* = 3) and uninfected mice (mock, red dot, *n* = 3) based on RNA-seq data. **b** Relative mRNA levels of *Trem2*, *Gfap*, *Map2*, *Syn2*, *Bace1 and Psen1* in hippocampal tissues of the mice at 30 dpi. The *β-actin* was used as a control for normalization during qRT-PCR. Mean ± SD; *n* = 3; *n.s.*, not significant; *, *p* < 0.05; **, *p* < 0.01, Student’s *t*-test.

**
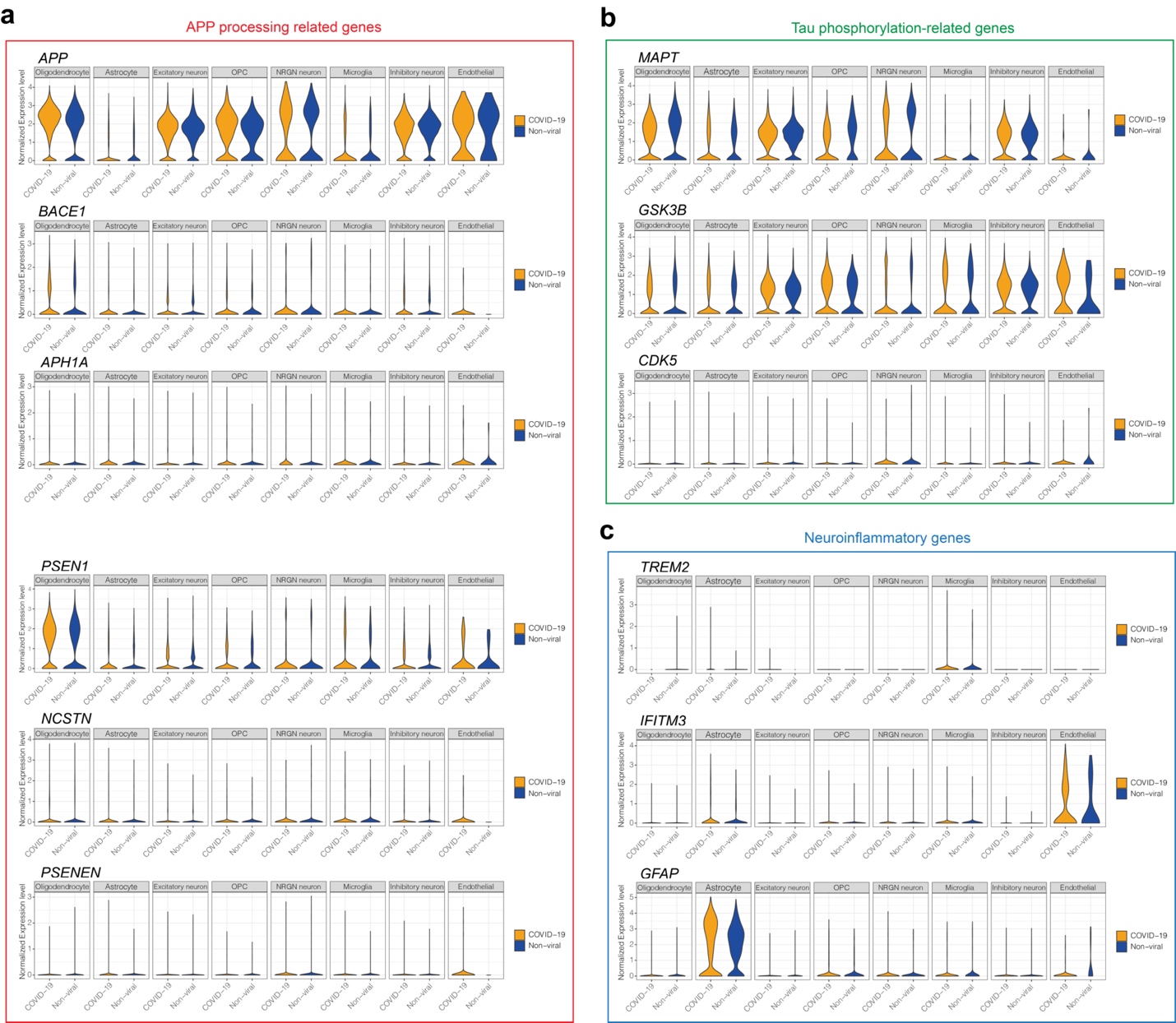
**

**Fig. S3 Upregulation of neuroinflammatory genes and un-alteration of APP processing- and tau phosphorylation-related genes in brain cells of the COVID-19 patients**

**a-c** The brain transcriptomic data^13^ (from single-nucleus RNA-seq) in COVID-19 patients was re-analyzed to show the expression pattern of the APP processing-related genes *APP*, *BACE1*, *APH1A*, *PSEN1*, *NCSTN* and *PSENEN* (a), tau phosphorylation-related genes *MAPT*, *GSK3B* and *CDK5* (**b**), and neuroinflammatory genes including *TREM2*, *IFITM3* and *GFAP* (**c**).


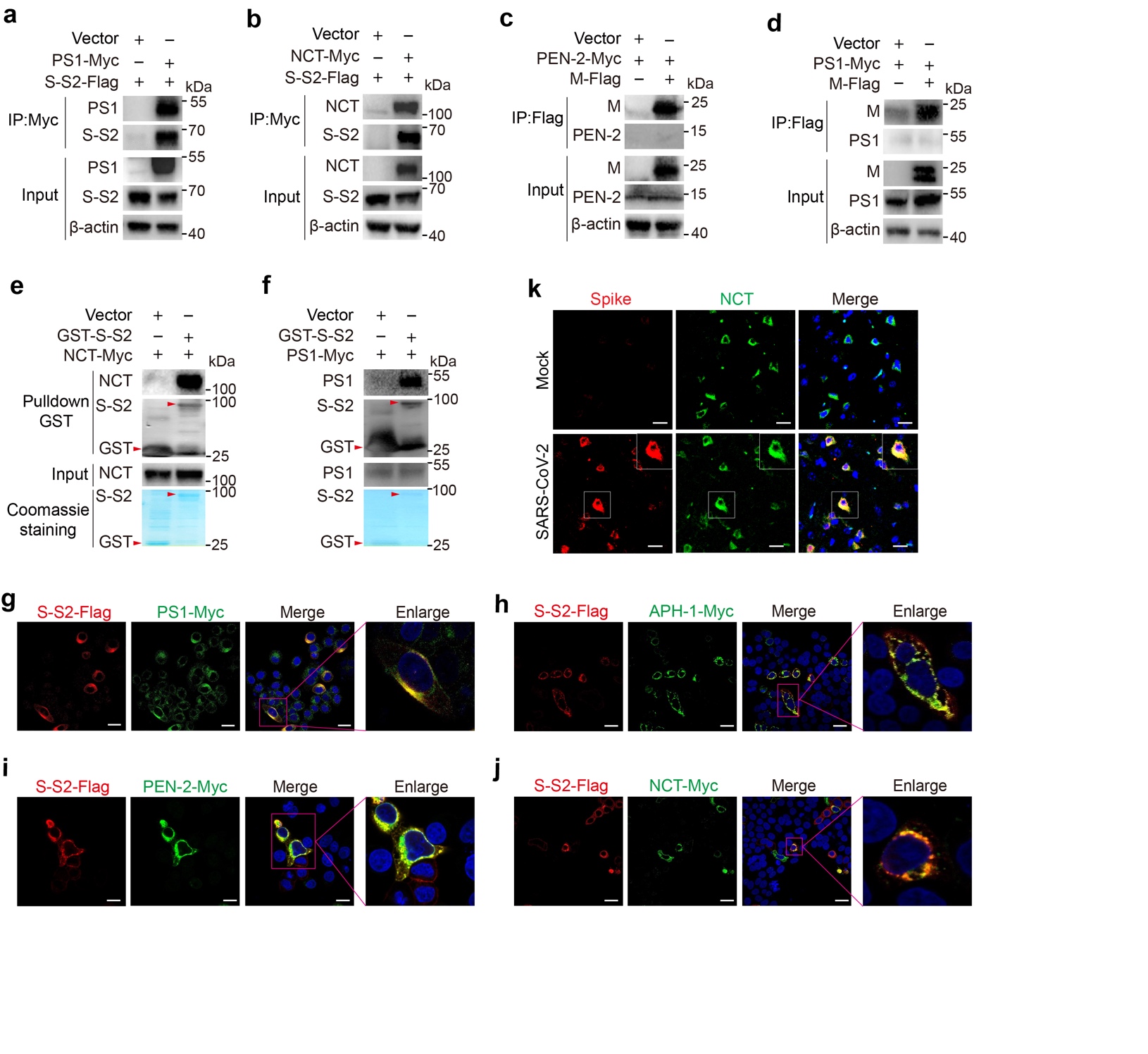


**Fig. S4 SARS-CoV-2 Spike S2 subunit binds to γ-secretase**

**a**,**b** co-IP assays of anti-myc monoclonal antibody in HEK293T cells transfected with myc-tagged PS1 (**a**) and myc-tagged NCT (**b**), together with flag-tagged prototyped S-S2 (S-S2-Falg) or empty vector (Vector). **c**,**d** co-IP assays of anti-flag monoclonal antibody in HEK293T cells transfected with flag-tagged expression vector for prototyped SARS-CoV-2 M protein (M-Flag) or empty vector (Vector), together with myc-tagged PEN-2 (**c**) or myc-tagged PS1 (**d**). **e**,**f** GST pulldown assays for bacterial GST (Vector) or GST-S-S2 incubated with cell lysates of HEK293T transfected with myc-tagged NCT (**e**) or PS1 (**f**). Coomassie blue staining and anti-GST and anti-myc blots were analyzed. **g-j** Representative immunofluorescence of exogenous prototyped flag-tagged S-S2 (S-S2-Flag) with myc-tagged PS1 (PS1-Myc, **g**), APH-1 (APH-1-Myc, **h**), PEN-2 (PEN-2-Myc, **i**), or NCT (NCT-Myc, **j**) in HeLa cells. Scale bar, 10 μm. **k** Representative immunofluorescence of endogenous SARS-CoV-2 Spike and NCT proteins in cortical sections of mice with or without SARS-CoV-2 infection at 7 dpi. Scale bar, 30 μm.

**
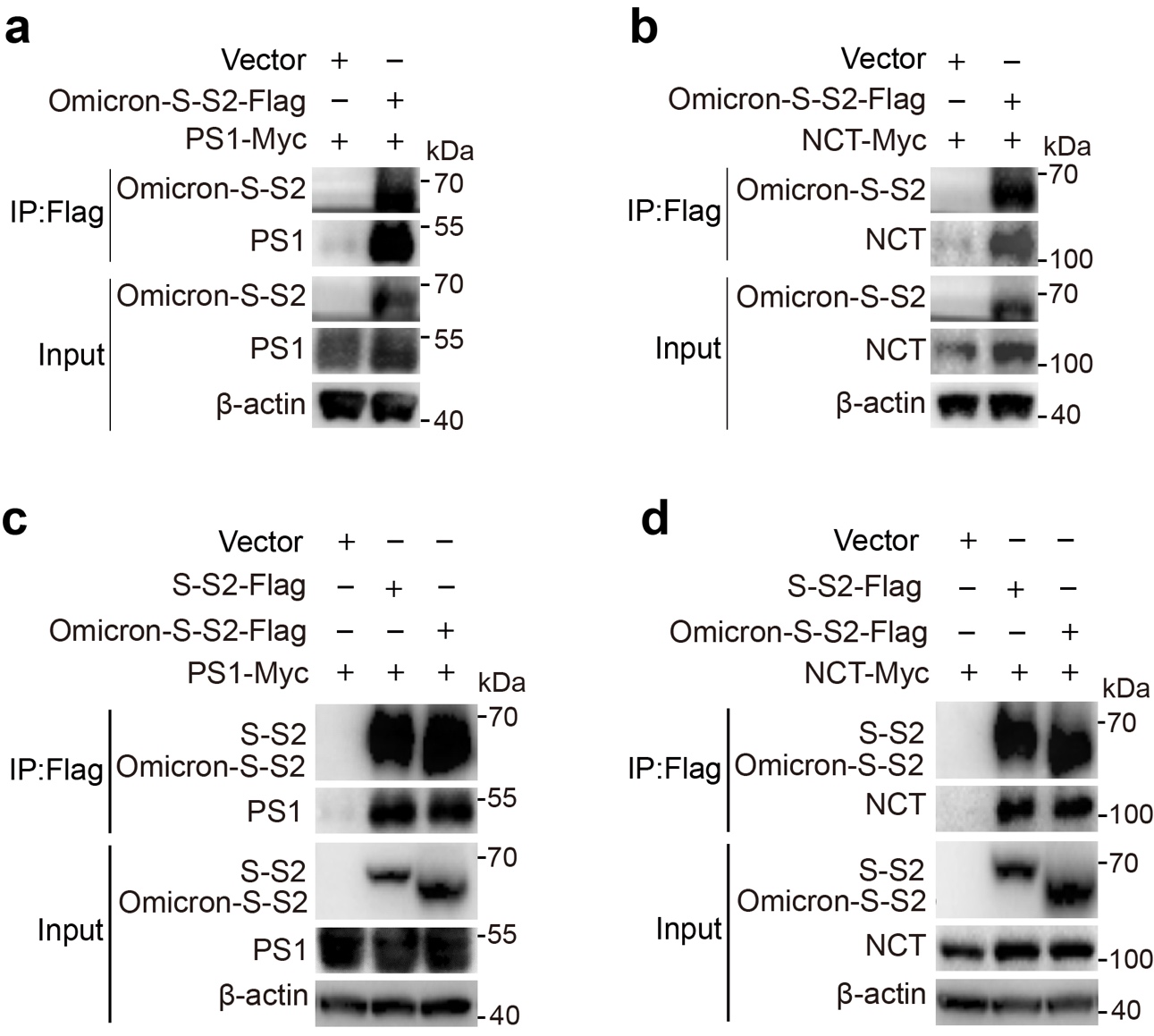
**

**Fig. S5 Omicron Spike S2 subunit binds to γ-secretase**

**a**,**b** co-IP assays of anti-flag monoclonal antibody in HEK293T cells transfected with flag-tagged Omicron Spike S2 subunit (Omicron-S-S2) or empty vector (Vector), together with myc-tagged PS1 (**a**) or NCT (**b**). **c**,**d** Direct comparison of the interaction of PS1 (**c**) or NCT (**d**) with S-S2 between the prototype and Omicron in same co-IP assay. Anti-flag and anti-myc blots were analyzed.

**
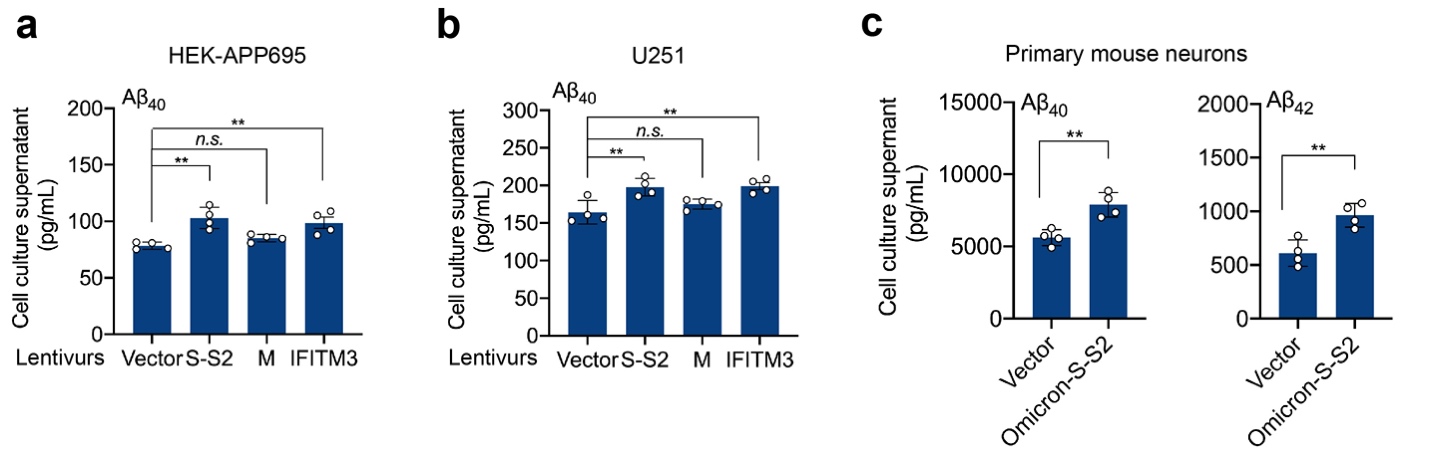
Fig. S6 Expression of SARS-CoV-2 Spike protein S2 subunit increases Aβ production**

**a** HEK293T cells stably expressing APP695 (HEK-APP695) were transfected with expression vector for prototyped S-S2, M, or IFITM3, or empty vector (Vector) (each 0.5 μg) in 24-well plates for 36 h. **b** U251 cells were transduced with lentivirus carrying prototyped S-S2, M, or IFITM3 in 24-well plates for 36 h. The Aβ40 level in the supernatants in **a** and **b** was quantified by ELISA. Mean ± SD; *n* = 4; *n.s.*, not significant; **, *p* < 0.01, one-way ANOVA with Bonferroni’s *post hoc* test. **c**, Quantification of the Aβ40 (*left*) and Aβ42 (*right*) levels by ELISA in the supernatants of mouse primary neurons transduced with lentivirus carrying Omicron-S-S2 for 36 h or empty vector (Vector). Mean ± SD; *n* = 4; **, *p* < 0.01, Student’s *t*-test.


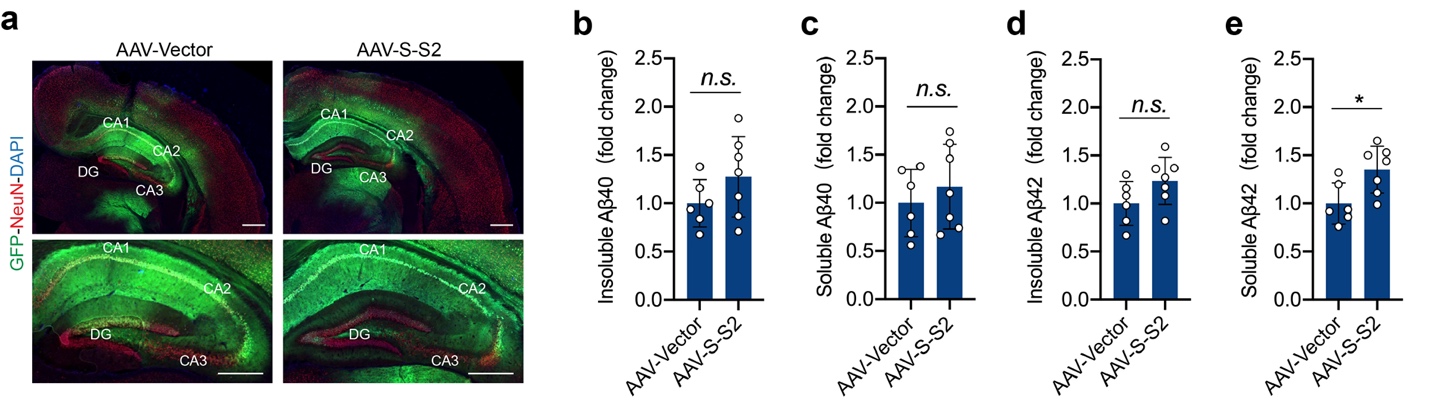


**Fig. S7 Overexpression of SARS-CoV-2 Spike S2 subunit via adeno-associated virus delivery** **increases Aβ deposit in APP/PS1ΔE9 mice**

**a** Fluorescence signals in brain sections of 7-month-old APP/PS1ΔE9 mice receiving AAV-Vector or AAV-S-S2 injection at age of 5 months old. Scale bar, 500 μm. **b-e** Quantification of the indicated insoluble Aβ40 and Aβ42, and soluble Aβ40 and Aβ42 in cortical tissues of the mice in **a** by ELISA. Mean ± SD; *n* = 6 or 7; *n.s.*, not significant; *, *p* < 0.05, Student’s *t*-test.


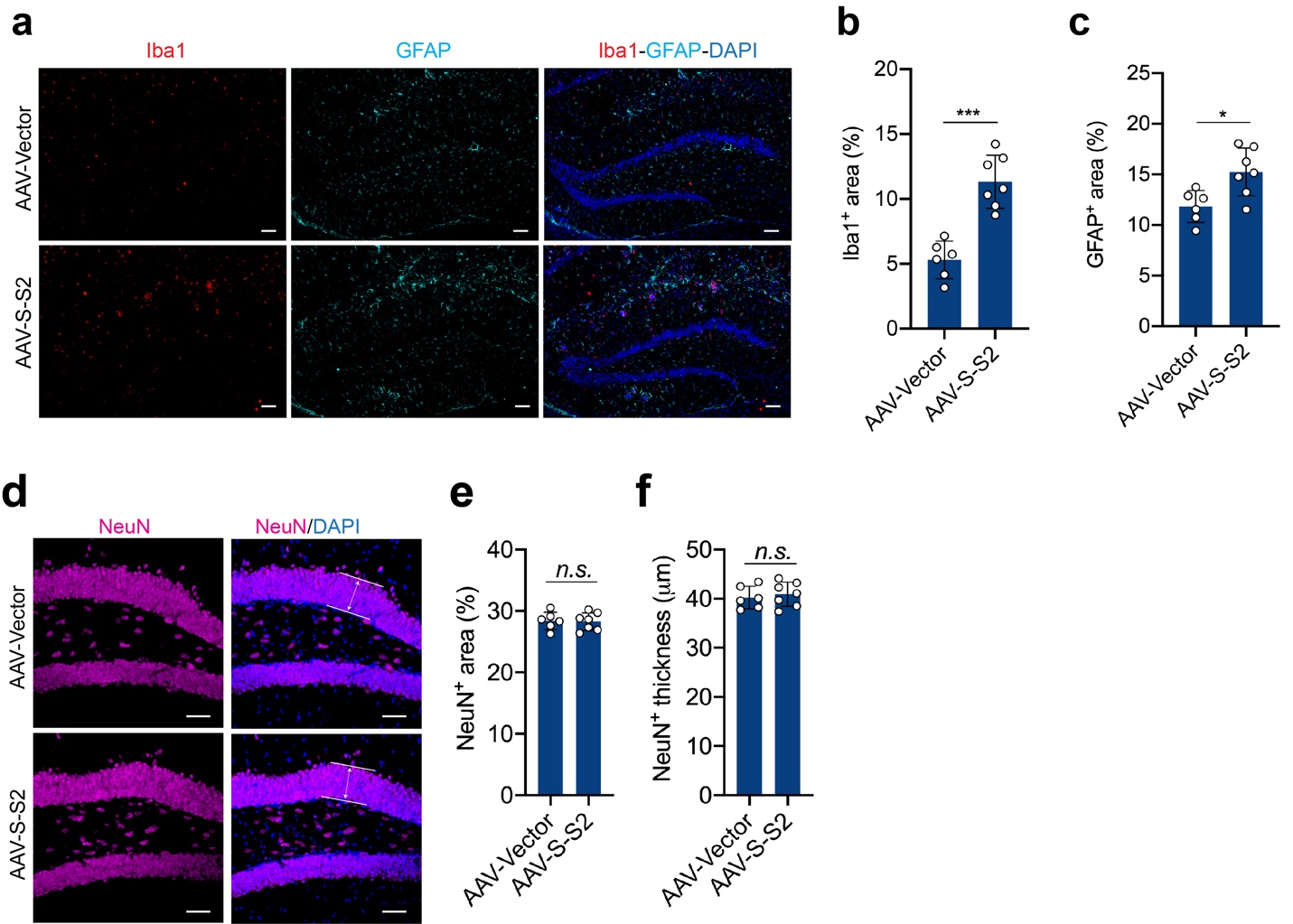


**Fig. S8 Overexpression of SARS-CoV-2 Spike S2 subunit causes the activation of glial cells in APP/PS1ΔE9 mice**

**a** Representative immunofluorescence of microglial Iba1 and astrocytic GFAP in hippocampal sections of APP/PS1ΔE9 mice with AAV delivery for SARS-CoV-2 S-S2 (AAV-S-S2) or AAV control (AAV-Vector). Scale bar, 30 μm. **b**,**c** Quantification of percentage of Iba1^+^ (**b**) and GFAP^+^ (**c**) area in **a**. **d** Representative immunofluorescence of NeuN protein in hippocampal sections of APP/PS1ΔE9 mice with AAV delivery for SARS-CoV-2 S-S2 (AAV-S-S2) or AAV control (AAV-Vector). Scale bar, 30 μm. **e**,**f** Quantification of percentage of NeuN^+^ area (**e**) and NeuN^+^ thickness (**f**) in dentate gyrus area in **d**. Each slide was used and counted for stained area via ImageJ software, and the percentage of average of stained area each section was calculated. Statistical analyses for **b**, **c**, **e** and **f**, Mean ± SD; *n* = 6 (AAV-Vector group) or *n* = 7 (AAV-S-S2 group); *n.s.*, not significant; *, *p* < 0.05; ***, *p* < 0.001, Student’s *t*-test.


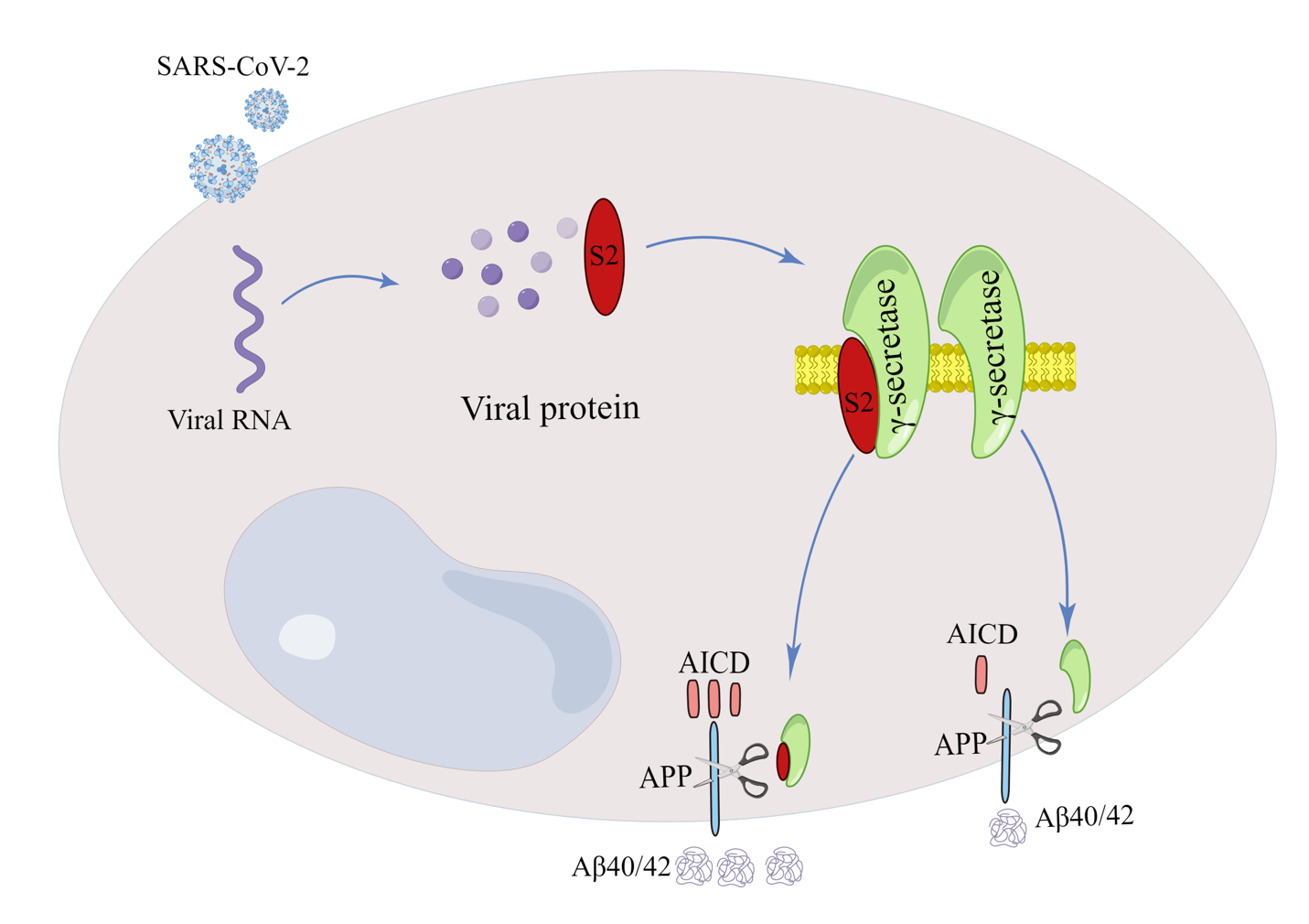


**Fig. S9 Diagram showing the mechanism of the current study (by Figdraw)**

Spike S2 subunit of SARS-CoV-2 infected cells interacts with γ-secretase and promotes its enzymatic cleavage of amyloid protein precursor (APP) to enhance the production of APP intracellular domain (AICD) and Aβ species, which contributed to a series of downstream consequences that finally cause neural cell dysfunction and neurodegeneration.
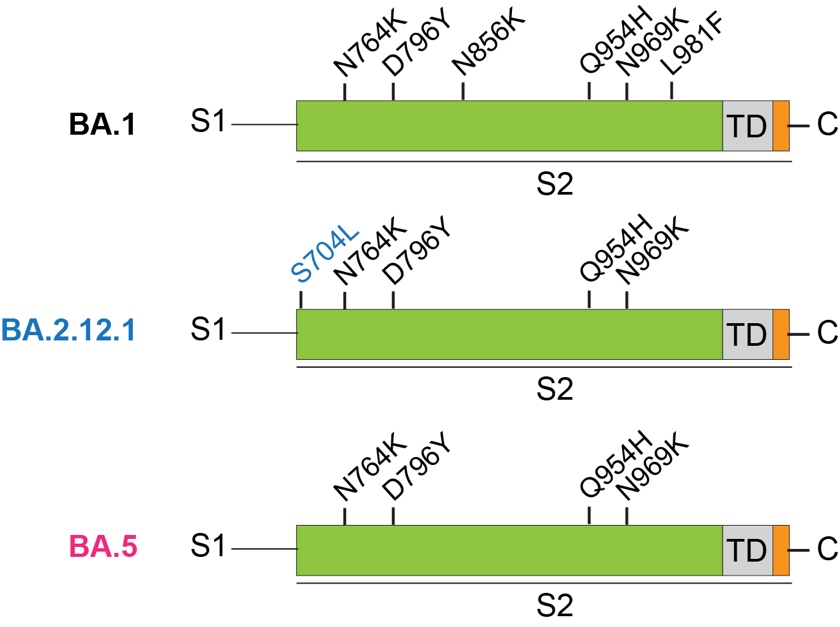


**Fig. S10 Diagram showing mutations in the Spike S2 subunit of the indicated Omicron sub-variants**.

**Supplementary References**

1 Zeng, J. *et al.* TRIM9-mediated resolution of neuroinflammation confers neuroprotection upon ischemic stroke in mice. *Cell Rep* **27**, 549-560, doi:10.1016/j.celrep.2018.12.055 (2019).

2 Song, T. Z. *et al.* Delayed severe cytokine storm and immune cell infiltration in SARS-CoV-2-infected aged Chinese rhesus macaques. *Zool Res* **41**, 503-516, doi:10.24272/j.issn.2095-8137.2020.202 (2020).

3 Xu, L. *et al.* COVID-19-like symptoms observed in Chinese tree shrews infected with SARS-CoV-2. *Zool Res* **41**, 517-526, doi:10.24272/j.issn.2095-8137.2020.053 (2020).

4 Chang, H. *et al.* Transient axonal glycoprotein-1 induces apoptosis-related gene expression without triggering apoptosis in U251 glioma cells. *Neural Regen Res* **9**, 519-525, doi:10.4103/1673-5374.130079 (2014).

5 Dilsizoglu Senol, A. *et al.* PAT1 inversely regulates the surface Amyloid Precursor Protein level in mouse primary neurons. *BMC Neurosci* **16**, 10, doi:10.1186/s12868-015-0152-8 (2015).

6 Zhang, D. F. *et al.* Complement C7 is a novel risk gene for Alzheimer's disease in Han Chinese. *Natl Sci Rev* **6**, 257-274, doi:10.1093/nsr/nwy127 (2019).

7 Zheng, H. Y. *et al.* Longitudinal transcriptome analyses show robust T cell immunity during recovery from COVID-19. *Sig Transduct Target Ther* **5**, 294, doi:10.1038/s41392-020-00457-4 (2020).

8 Bolger, A. M., Lohse, M. & Usadel, B. Trimmomatic: a flexible trimmer for Illumina sequence data. *Bioinformatics* **30**, 2114-2120, doi:10.1093/bioinformatics/btu170 (2014).

9 Dobin, A. *et al.* STAR: ultrafast universal RNA-seq aligner. *Bioinformatics* **29**, 15-21, doi:10.1093/bioinformatics/bts635 (2013).

10 Li, B. & Dewey, C. N. RSEM: accurate transcript quantification from RNA-Seq data with or without a reference genome. *Bmc Bioinformatics* **12**, 323, doi:10.1186/1471-2105-12-323 (2011).

11 Love, M. I., Huber, W. & Anders, S. Moderated estimation of fold change and dispersion for RNA-seq data with DESeq2. *Genome Biol* **15**, 550, doi:10.1186/s13059-014-0550-8 (2014).

12 Yu, G. C., Wang, L. G., Han, Y. Y. & He, Q. Y. clusterProfiler: an R Package for Comparing Biological Themes Among Gene Clusters. *Omics* **16**, 284-287, doi:10.1089/omi.2011.0118 (2012).

13 Yang, A. C. *et al.* Dysregulation of brain and choroid plexus cell types in severe COVID-19. *Nature* **595**, 565-571, doi:10.1038/s41586-021-03710-0 (2021).

14 Butler, A., Hoffman, P., Smibert, P., Papalexi, E. & Satija, R. Integrating single-cell transcriptomic data across different conditions, technologies, and species. *Nat Biotechnol* **36**, 411-420, doi:10.1038/nbt.4096 (2018).

15 Zeng, J. *et al.* Specific inhibition of the NLRP3 inflammasome suppresses immune overactivation and alleviates COVID-19 like pathology in mice. *eBioMedicine* **75**, 103803, doi:10.1016/j.ebiom.2021.103803 (2022).

16 Zeng, J. *et al.* The zika virus capsid disrupts corticogenesis by suppressing dicer activity and miRNA biogenesis. *Cell Stem Cell* **27**, 618-632, doi:10.1016/j.stem.2020.07.012 (2020).

17 Xie, X. C. *et al.* Emerging SARS-CoV-2 B.1.621/Mu variant is prominently resistant to inactivated vaccine-elicited antibodies. *Zool Res* **42**, 789-791, doi:10.24272/j.issn.2095-8137.2021.343 (2021).

18 Jankowsky, J. L. *et al.* Mutant presenilins specifically elevate the levels of the 42 residue beta-amyloid peptide in vivo: evidence for augmentation of a 42-specific gamma secretase. *Hum Mol Genet* **13**, 159-170, doi:10.1093/hmg/ddh019 (2004).

19 Luo, R. *et al.* A novel missense variant in ACAA1 contributes to early-onset Alzheimer's disease, impairs lysosomal function, and facilitates amyloid-beta pathology and cognitive decline. *Signal Transduct Target Ther* **6**, 325, doi:10.1038/s41392-021-00748-4 (2021).

20 Luo, R. *et al.* Activation of PPARA-mediated autophagy reduces Alzheimer disease-like pathology and cognitive decline in a murine model. *Autophagy* **16**, 52-69, doi:10.1080/15548627.2019.1596488 (2020).

21 Piller, C. Blots on a field? *Science* **377**, 358-363, doi:10.1126/science.add9993 (2022).

22 Zott, B. *et al.* A vicious cycle of beta amyloid-dependent neuronal hyperactivation. *Science* **365**, 559-565, doi:10.1126/science.aay0198 (2019).

23 Pascoal, T. A. *et al.* Microglial activation and tau propagate jointly across Braak stages. *Nat Med* **27**, 1592-1599, doi:10.1038/s41591-021-01456-w (2021).

24 Karran, E. & De Strooper, B. The amyloid hypothesis in Alzheimer disease: new insights from new therapeutics. *Nat Rev Drug Discov* **21**, 306-318, doi:10.1038/s41573-022-00391-w (2022).

25 Palop, J. J. & Mucke, L. Amyloid-beta-induced neuronal dysfunction in Alzheimer's disease: from synapses toward neural networks. *Nat Neurosci* **13**, 812-818, doi:10.1038/nn.2583 (2010).
